# Regulatory QTLs affecting miRNA-mRNA interactions in cancer: mechanisms, methods, and clinical implications

**DOI:** 10.3389/fmolb.2025.1749396

**Published:** 2026-01-15

**Authors:** Vivek Kumar, Rohit Shukla, Amit Chaudhary, Mansi Gautam

**Affiliations:** 1 USF Center for Microbiome Research, Microbiomes Institute, University of South Florida, Tampa, FL, United States; 2 Heersink School of Medicine, Department of Cell, Developmental and Integrative Biology, The University of Alabama at Birmingham, Birmingham, AL, United States; 3 Department of Biomedical Engineering, Galgotias University, Noida, Uttar Pradesh, India

**Keywords:** biomarker, cancer, eQTL, miRNA, regQTLs, SNP

## Abstract

MicroRNAs (miRNAs) are post-transcriptional regulators that play essential roles in cancer initiation, progression, and therapy response. Single nucleotide polymorphisms (SNPs) that affect miRNA-mRNA interactions, termed regulatory quantitative trait loci (regQTLs), have emerged as critical modulators of gene expression landscapes in tumors. These regQTLs can disrupt or enhance miRNA binding to target sites, modulate transcript stability, and influence oncogenic or tumor-suppressive pathways, thus shaping individual cancer susceptibility and clinical outcomes. In this review, we comprehensively examine the biological, computational, and translational aspects of regQTLs in cancer. We summarize key computational approaches used to investigate germline influences on miRNA-mediated regulation, including interaction-based regQTL models, miR- and isomiR-eQTL analyses, and sequence-based prediction tools. We further discuss emerging miRNA-TWAS methods, which do not directly detect regQTLs but provide a valuable upstream strategy by identifying genetically regulated miRNAs that may participate in downstream regQTL interactions. We also summarize publicly available datasets and annotation platforms supporting large-scale discovery efforts. Through critical evaluation of recent experimental validations and clinical association studies, we highlight regQTLs that serve as biomarkers for prognosis and therapy response in diverse cancers such as breast, lung, prostate, and colorectal. Furthermore, we explore the therapeutic potential of targeting miRNA–SNP interactions, including emerging strategies in miRNA-tailored immunotherapies and mRNA vaccines. We propose a strategic roadmap for future research, emphasizing the need for population-specific analyses, single-cell regQTL mapping, and mechanistic dissection using multi-omic models. By connecting genetic variation, regulatory biology, and clinical translation, this review provides a foundational framework to harness miRNA-regulatory QTLs for precision oncology.

## 1 Introduction

MicroRNAs (miRNAs) are small non-coding RNAs of approximately 22 nucleotides. They regulate gene expression by binding to complementary sequences within the 3′untranslated regions (3′-UTRs) of target mRNAs. This interaction typically results in translational repression or degradation of the mRNA molecule. These molecules regulate more than 60% of protein-coding genes at the post-transcriptional level. They play essential roles in key biological processes, including development, apoptosis, cell proliferation, and oncogenesis (Catalanotto et al., 2016). Dysregulated expression of miRNA has been reported in several cancer type not limited to ovarian, breast, colorectal and lung cancer (Kumar et al., 2019; Kumar et al., 2021; Kumar et al., 2022; Kumar et al., 2023a; Kumar et al., 2023b; Jafarzadeh et al., 2022).

Genetic variation, particularly single nucleotide polymorphisms (SNPs) play a crucial role in cancer susceptibility and progression. Although genome-wide association studies (GWAS) have identified thousands of cancer-associated SNPs, a large proportion of these variants reside in noncoding regions. Because they do not alter protein sequences, their mechanisms of action are often difficult to interpret. A subset of these variants is now known to influence miRNA-mediated regulation by either altering miRNA expression or disrupting miRNA-mRNA interactions. For example, SNPs located within miRNA seed regions, precursor sequences, or mRNA 3′-UTRs can modify the efficiency of post-transcriptional regulation (Mohamed and Freude, 2024).

To systematically evaluate these relationships, investigators have introduced the concept of regulatory quantitative trait loci (regQTLs), which describe SNPs that modulate the relationship between miRNA abundance and target mRNA expression in an allele-specific manner. Importantly, regQTLs do not represent a separate QTL class; rather, they overlap with existing post-transcriptional QTL categories such as 3′UTR-eQTLs and miR-eQTLs. What distinguishes regQTL analysis is its focus on SNP × miRNA × mRNA interaction effects, enabling the detection of allelic variants that alter miRNA-mediated repression within tumors. Using this analytical framework, Wilk and Braun (2018b) identified numerous SNPs across breast, liver, lung, and prostate cancers that modify miRNA–mRNA regulatory relationships (Wilk and Braun, 2018b).

In parallel, genome-wide studies in both healthy and disease cohorts have mapped miRNA expression quantitative trait loci (miR-eQTLs). These loci represent SNPs that are associated with variation in the expression levels of mature miRNAs. Huan et al. (2015) reported over 5,200 such cis-miR-eQTLs in whole blood samples from more than 5,000 individuals, several of which overlapped known disease loci (Huan et al., 2015). More recently, Mustafa et al. (2024) identified over 4,300 miR-eQTLs across 64 miRNAs, many of which co-localize with cancer susceptibility regions (Mustafa et al., 2024).

Further, isomiR-eQTL analyses was conducted to evaluate variants that influence alternative forms of miRNAs, have revealed tens of thousands of SNPs that modulate miRNA isoform expression across cancer types (Moradi et al., 2022). These findings underscore the vast regulatory impact of noncoding SNPs on miRNA-mediated gene control, although many results remain computational and lack experimental follow-up.

Despite these advancements, no dedicated review has yet addressed the growing body of work on regQTLs in cancer. Previous reviews have broadly discussed SNPs in miRNA genes or target sites, but not the broader framework of allele-specific modulation of miRNA–mRNA regulatory interactions in tumors (Preskill and Weidhaas, 2013). Given the increasing recognition of post-transcriptional regulation in cancer biology, and the emerging role of noncoding SNPs in shaping gene expression networks, this review aims to fill this gap. We provide a comprehensive overview of regulatory QTLs affecting miRNA activity in cancer, summarizing key discoveries, tools, mechanistic insights, and clinical relevance.

## 2 Molecular mechanisms of regulatory QTLs in cancer

Recent research has revealed that genetic variants can modulate the interaction between miRNAs and their target mRNAs, adding a previously underappreciated layer of post-transcriptional regulatory complexity in cancer biology. These variants are referred to as regulatory QTLs (regQTLs), which represent a functional subset of noncoding regulatory variants that influence miRNA–mRNA interactions. Rather than constituting a separate or unique class, regQTLs partially overlap with established QTL categories—including 3′UTR-eQTLs, miR-eQTLs, and other post-transcriptional eQTLs—yet are distinguished by their mechanistic focus on allele-dependent modulation of miRNA binding or target accessibility.

Through these mechanisms, regQTLs alter gene expression by changing miRNA binding efficacy, miRNA availability, or the local structural environment of the mRNA in an allele-specific manner. The resulting SNP-driven variation may exert subtle or profound regulatory impact depending on the biological function of the affected target gene and the surrounding tumor microenvironment context (Li et al., 2023; Tian et al., 2024; Bhatia et al., 2024).

### 2.1 Molecular origins and types of regQTLs

Regulatory QTLs that affect miRNA–mRNA interactions represent a functional subset of noncoding regulatory variants, rather than a completely unique class. These variants often overlap with established categories such as 3′UTR-eQTLs, miR-eQTLs, and post-transcriptional eQTLs, but are distinguished by their mechanistic effect on allele-specific modulation of miRNA binding, target accessibility, or transcript stability. These SNPs can alter miRNA binding sites, miRNA expression, or the structural accessibility of 3′UTRs and coding regions, thereby rewiring post-transcriptional regulatory networks in cancer. Understanding their molecular origins and classification is essential for interpreting their roles in tumorigenesis and therapeutic response (Wilk and Braun, 2018b). The Most common molecular source of regQTLs is listed below.

#### 2.1.1 3′UTR SNPs in target genes

**FIGURE 1:**
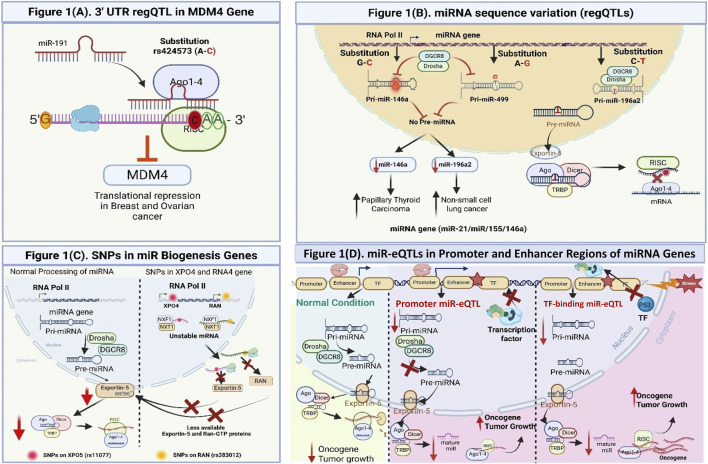
Spectrum of regulatory QTLs (regQTLs) influencing miRNA expression, processing, and oncogenic activation. **(A)** 3′UTR regQTL (rs424573 A→C) in the *MDM4* gene creates a novel miR-191 binding site, facilitating AGO1-4-RISC–mediated translational repression and enhanced p53 activation in breast and ovarian cancer. **(B)** SNPs within miRNA genes, including rs2910164 (G→C) in *miR-146a*, rs11614913 (C→T) in *miR-196a2*, and rs3746444 (A→G) in *miR-499*, alter stem-loop conformation and Drosha/Dicer processing efficiency, resulting in reduced mature miRNA levels and increased tumor risk. **(C)** regQTLs in miRNA biogenesis genes, such as rs11077 in *XPO5* and rs3803012 in *RAN*, destabilize their mRNAs, decreasing Exportin-5 and Ran-GTP proteins and impairing pre-miRNA nuclear export, which limits cytoplasmic Dicer processing and mature miRNA formation. **(D)** miR-eQTLs in Promoter and Enhancer Regions of miRNA Genes. Promoter-, enhancer-, and transcription-factor–binding miR-eQTLs regulate the transcriptional output of miRNA genes by altering chromatin accessibility, enhancer–promoter looping, and recruitment of RNA Polymerase II or stress-activated transcription factors such as p53. Genetic variants that weaken enhancer activity, reduce promoter occupancy, or disrupt TF binding lead to decreased pri-miRNA transcription, reduced processing by Drosha/DGCR8, and diminished production of mature miRNAs. These variants influence miRNA abundance, rather than miRNA–mRNA interaction strength and therefore represent miR-eQTLs rather than regQTLs. By altering upstream transcriptional control, miR-eQTLs can indirectly contribute to derepression of oncogenic targets and promote tumor progression.

The presence of SNPs in 3′-UTRs of target mRNAs can disrupt or create miRNA recognition elements (MREs). These MREs weaken or enhance the miRNA binding thus resulting in upregulated or downregulated expression of target gene. Such variants directly alter the affinity or accessibility of the binding site (Hajibabaie et al., 2022). Mechanistically, SNPs in the 3′UTR affect miRNA–mRNA regulation by modifying complementarity within the seed region or altering the secondary structure of the mRNA that governs miRNA accessibility. These changes influence the thermodynamic stability (ΔG) of the miRNA–target duplex and the recruitment of AGO2 within the RISC complex. Loss-of-function variants lead to decreased RISC loading and derepression of oncogenic transcripts, whereas gain-of-function alleles can enhance silencing of tumor suppressors. These molecular shifts underline allelic imbalance in gene expression and contribute to inter-individual variation in tumor susceptibility (Saunders et al., 2007). The detailed mechanism is outlined in Figure 1A.

Recent studies have shown that alleles that interfere with seed pairing are capable of inhibiting the corresponding miRNA’s regulation, that can end up with oncogene depression. For instance, rs4245739 in the MDM4 3′UTR modifies breast cancer susceptibility by creating a unique binding site for miR-191 (Jacinta-Fernandes et al., 2020). Similarly, another study revealed that *KRAS* 3′UTR with SNPs T>G (rs61764370) disrupts the complementary pairing with the tumor-suppressive let-7 miRNA. Which results in loss of translational repression and enhanced KRAS signaling promoting oncogenic transformation (Chin et al., 2008). Another functionally validated variant is rs16917496 (T>C) within the *SET8/KMT5A* 3′UTR. These variants reside in the miR-502 recognition site. The presence of C allele reduces binding efficiency of miR-502. Which further increases the expression of SET8, histone H4K20 methylation, and dysregulated cell-cycle progression (Song et al., 2009). Collectively, these 3′UTR regQTLs demonstrate how single-base alterations may remodel miRNA–mRNA interaction, that changes the oncogenic signaling and increases tumor heterogeneity.

#### 2.1.2 RNA modification–based molecular mechanisms in miRNA regulation

Epitranscriptomic RNA modifications constitute a major regulatory layer shaping RNA metabolism independently of the underlying nucleotide sequence. More than 170 chemical RNA modifications have been identified across coding and non-coding RNAs, with N^6^-methyladenosine (m6A) representing the most abundant and best-characterized internal mark in mRNA (Xuan et al., 2024). The m6A mark is dynamically added by the METTL3–METTL14–WTAP “writer” complex and interpreted by “reader” proteins such as HNRNPA2B1, YTH family members, and IGF2BP proteins. Through these interactions, RNA modifications influence diverse post-transcriptional processes including RNA stability, alternative splicing, nuclear export, and translation (Berulava et al., 2020; Chen et al., 2021; Jiang et al., 2021).

Because these processes determine RNA maturation, localization, and lifespan, epitranscriptomic modifications have substantial potential to affect miRNA pathways indirectly. They may alter the abundance of primary miRNA transcripts, influence their processing efficiency, or modify target mRNA accessibility. These contributions introduce an additional, reversible layer of regulation that can interact with allele-specific mechanisms captured by regQTLs, particularly in cancer settings where RNA modification enzymes are frequently dysregulated (Mei et al., 2023).

##### 2.1.2.1 m6A as an epitranscriptomic regulator of miRNA–mRNA interactions

Among RNA modifications, m6A stands out as a major epitranscriptomic regulator of post-transcriptional gene expression with direct relevance to miRNA biology. m6A marks are deposited by METTL3–METTL14 and removed by FTO and ALKBH5, while reader proteins such as HNRNPA2B1, YTHDF proteins, and IGF2BP family members interpret the modification to guide RNA fate (Petri and Klinge, 2023). Through these dynamic interactions, m6A influences miRNA maturation, target accessibility, and the efficiency of RISC-mediated repression. A well-established mechanism involves the role of m6A in miRNA biogenesis. Alarcón et al. demonstrated that specific pri-miRNAs contain m6A marks recognized by the nuclear reader HNRNPA2B1, which recruits DGCR8 to enhance Microprocessor-mediated cleavage. Knockdown of METTL3 reduces m6A levels on pri-miRNAs and impairs their processing, while depletion of HNRNPA2B1 similarly disrupts DGCR8 recruitment, confirming that m6A functions as a biochemical determinant of miRNA maturation (Alarcón et al., 2015). Because regQTLs rely on differences in the abundance of mature miRNAs to modulate allele-specific target repression, m6A-driven changes in Microprocessor efficiency represent a key regulatory intersection.

Moreover, m6A also shapes the structural landscape of mRNAs, which may indirectly influence miRNA target-site accessibility. The “m6A-switch” mechanism, described by Liu et al., showed that m6A destabilizes local RNA secondary structures, exposing single-stranded regions and facilitating interaction with RNA-binding proteins such as HNRNPC (Liu et al., 2015). While the direct impact of this structural remodeling on miRNA recognition elements (MREs) remains to be experimentally demonstrated, the enrichment of m6A sites near 3′-UTRs suggests that m6A could influence miRNA–mRNA interactions by altering RNA topology without requiring sequence changes (Zhu et al., 2020; Qian et al., 2021).

Recent studies also suggest that mature miRNAs may themselves undergo m6A modification, influencing their intracellular activity. Garbo et al. reported that m6A-modified miRNAs display reduced affinity for AGO2, decreased silencing efficiency, and are preferentially exported in extracellular vesicles (Garbo et al., 2024). Although further validation is needed, these findings highlight an additional reversibility mechanism in miRNA regulation. Overall, the regulatory significance of m6A in miRNA pathways is underscored by the discovery of m6A-QTLs, genetic variants that modulate m6A deposition in transcripts. Xiong et al. identified m6A-QTLs across multiple human tissues and demonstrated that many colocalize with disease-associated regulatory loci, including cancer-related regions (Xiong et al., 2021). When such variants occur near pri-miRNAs or in 3′-UTRs containing MREs, they may alter local methylation patterns, thereby influencing miRNA maturation or binding affinity. This suggests that certain regQTL signals may be mediated—or modified—by epitranscriptomic variation rather than sequence change alone.

Collectively, these findings establish m6A as a central determinant of miRNA function through its effects on miRNA biogenesis, target accessibility, and RISC engagement. In cancer, where m6A regulators are frequently dysregulated, the integration of epitranscriptomic profiling into regQTL analyses is likely to enhance mechanistic interpretation and reveal previously unrecognized regulatory interactions that contribute to tumor heterogeneity.

#### 2.1.3 miRNA sequence variants and their functional consequences in cancer

Another kind of genetic polymorphism is present within the miRNA gene sequences. Which includes the primary (pri-miRNA), precursor (pre-miRNA), and mature miRNA regions. These variations are emerging as crucial regulators of miRNA biogenesis, stability, and target interaction specificity. Moreover, these variants act as functional regulatory variants, that significantly influence cancer risk and progression by disrupting the precise post-transcriptional regulatory functions of miRNAs (Liu et al., 2025). Mechanistically, the miRNA sequence variation itself disrupts the biogenesis pathway at different stages. The variation in pri-miRNAs sequence alters the transcription efficiency as well as processing by the Drosha–DGCR8 complex. Similarly, changes in pre-miRNA stem-loop stability can influence nuclear export through Exportin-5/Ran-GTP. Moreover, these variations also affect the cytoplasmic cleavage by Dicer. In addition, the mutations within the mature miRNA sequence influence the seed-region complementarity, that further alters the target specificity and binding affinity (ΔG). These structural and thermodynamic changes can alter the expression patterns of oncogenes or tumor-suppressors by causing allele-specific variations in miRNA abundance or targeting efficacy (Fernandez et al., 2017). The mechanism is illustrated in Figure 1B.

One of the most extensively studied sequence variants is rs2910164 (G>C) located in the *miR-146a* precursor. This SNP alters the stem-loop structure of the pre-miRNA, resulting in reduced processing efficiency by Drosha and Dicer, and consequently decreased levels of mature miR-146a. Functional studies have shown that this dysregulation contributes to increased susceptibility to papillary thyroid carcinoma and other malignancies through impaired negative regulation of pro-inflammatory signaling pathways (Jazdzewski et al., 2008). Similarly, the rs11614913 (C>T) polymorphism in *miR-196a2*, located within the mature sequence, has been shown to affect Dicer processing and miRNA expression levels. The T allele is associated with increased risk for non-small cell lung cancer and reduced overall survival in affected individuals. This variation possibly affects miRNA-target interactions and modifies the thermodynamic stability of the miRNA duplex (Yuan et al., 2013b). Similarly, the stem-loop region of mature miR-499 sequence have functionally relevant variation rs3746444 (A>G). The variation has been linked to poor maturation and altered precursor folding. Several recent meta-analyses have linked the G allele with a modest but statistically significant increase in overall risk of hepatocellular, breast, and gastric cancers among Asian populations (Qiu et al., 2012).

Collectively, these results highlight the significant impact of sequence variations within miRNA genes on their synthesis and regulatory capacity. Therefore, these polymorphisms offer mechanistic insights as well as biomarker capacity to assess cancer risk.

#### 2.1.4 SNPs in miRNA biogenesis genes

Precise miRNA biosynthesis is required for maintenance of post-transcriptional regulation of gene expression. This process is orchestrated by a conserved set of enzymes and transport proteins. This process is series of chained event where enzymes like Drosha, DGCR8, Exportin-5 (XPO5), RAN, DICER and RISC complex are involved in different compartment of cell. In the nucleus, the Drosha–DGCR8 complex cleaves pri-miRNAs into precursor hairpins. These pre-miRNAs are further exported to the cytoplasm via the XPO5–RAN–GTP complex. Then pre-miRNA is processed by Dicer into ∼22-nucleotide duplexes that guide mRNA silencing through the RISC complex (Yi et al., 2003; Denli et al., 2004; Ha and Kim, 2014). Therefore, alterations in any of these components can disrupt global miRNA homeostasis that leads to aberrant expression of oncogenes or tumor suppressors. Recent study suggested that reduced level of Dicer or Drosha expression are linked with poor prognosis and metastasis. In addition, impaired XPO5 leads to decrease the level of mature miRNA that results in transcriptional imbalance. Thus, polymorphisms or mutations in miRNA-processing genes can give rise to widespread miR-eQTL and regQTL effects, reprogramming miRNA landscapes and contributing to cancer initiation, progression, and immune evasion.

Recent studies suggested that regQTLs can also arise from polymorphisms present in core miRNA biogenesis genes including Drosha, Dicer, XPO5, and RAN. Study by Wojcikiewicz et al., on laryngeal cancer revealed the polymorphism withing the miRNA biogenesis molecules. Their findings suggest polymorphisms on DROSA (rs6877842), DGCR8 (RS3757, rs417309, rs1640299, RAN (rs14035), XPO5 (rs11077), DICER1 (rs13078 and rs3742330) and *TARBP2* (rs784567) were evidently present. Moreover, these variations are significantly correlated with the disruption in miRNA maturation and increased cancer susceptibility (Osuch-Wojcikiewicz et al., 2015). Similarly, another study by Yuan et al. found 3′UTR of DROSHA have T>C polymorphism (rs10719) and was associated with increased risk of bladder cancer (Yuan et al., 2013a). In another study, Shao et al. (2020) conducted a Bayesian meta-analysis revealing two key SNPs like rs11077 in *XPO5* and rs3803012 in *RAN*, that modulate cancer risk across populations. These variants impair nuclear export of pre-miRNAs, leading to dysregulated mature miRNA profiles. Even though these polymorphisms are not within miRNA genes, however, they act as indirect regQTLs that influence global post-transcriptional regulation and contributing to cancers (Shao et al., 2020). The mechanism by which these SNPs affect the biogenesis and global homoeostasis of miRNA is illustrated in Figure 1C.

#### 2.1.5 miR-eQTLs in promoter and enhancer regions of miRNA genes

Regulatory SNPs in promoter and enhancer regions of miRNA genes are frequently observed in several studies. These SNPs can modulate transcription by altering transcription factor binding or chromatin accessibility. These cis-acting variants, or cis-miR-eQTLs, influence the primary expression levels of miRNAs, thereby indirectly affecting downstream gene regulation. The molecular mechanism associates the presence of miR-eQTLs in the promoter- and enhancer-associated sequences of miRNA. These miR-eQTLs are involved in altering the binding of TF, chromatin accessibility and enhancer-promoter communication, thus leading to dysregulated expression of miRNA. Moreover, the variation in sequences within promoter motifs can disrupt consensus sites for regulators such as p53, MYC, or NF-κB. Thereby modulating RNA polymerase II recruitment and pri-miRNA transcription. Likewise, enhancer SNPs can influence histone acetylation (H3K27ac) or CTCF-mediated looping, changing enhancer–promoter interaction strength. These allele-specific effects lead to differential transcription of tumor-suppressive or oncogenic miRNAs, producing downstream dysregulation of mRNA targets. The regulatory mechanism is illustrated in Figure 1D.

In a genome-wide analysis, Huan et al. (2015) identified over 5,200 significant cis-miR-eQTLs in whole blood from >5,000 individuals, many of which mapped to promoter regions of cancer-associated miRNAs such as *miR-21*, *miR-155*, and *miR-146a* (Huan et al., 2015). A couple of these variations overlapped with identified GWAS loci. These imply that genetic risk for cancer and other complex disorders may be mediated by aberrant miRNA transcription. These results demonstrate the importance of promoter/enhancer-associated miR-eQTLs as another level of miRNA regulation that complements those in biogenesis or targeting pathways. In order to comprehensively map such regulatory variation, Wilk and Braun (2018b) developed a statistical framework that combines mRNA expression, miRNA expression, and germline genotype data to identify allele-specific modulation of miRNA–mRNA interactions. Based on TCGA dataset from different disease-like breast, lung, liver and prostate cancer, thousands of regQTLs were discovered that changes the intensity of miRNA–mRNA correlations based on patient’s genotype. These results offered compelling proof that noncoding variations can alter tumor transcriptomes via miRNA-related processes (Wilk and Braun, 2018b).

Based on this foundation, Moradi et al. (2022) identified approximately 150,000 miR-eQTLs and 2.4 million isomiR-eQTLs across 30 TCGA tumor types in a pan-cancer investigation of isomiR expression QTLs (isomiR-eQTLs). These included variations that particularly changed the quantity of non-canonical miRNA isoforms, which frequently demonstrated roles exclusive to cancer types. Numerous variations that were found overlapped with established GWAS loci. This supports the theory that miR-eQTLs may increase hereditary cancer susceptibility by modifying post-transcriptional regulation (Moradi et al., 2022). In addition, further complexity was introduced by adding exposure dependent eQTLs (e^2^QTLs), where immune cells were treated with DNA-damaging agents. Their research revealed that certain SNPs only control gene expression in stressful situations, especially in apoptosis and p53 signaling pathways. This suggests that the effects of eQTLs may be highly context-dependent and impacted by the tumor microenvironment or therapeutic pressure (Bigge et al., 2024). Wang and Hon (2024) simultaneously highlighted the necessity of better annotation of noncoding cancer variations, such as those in miRNA loci and their regulatory relationships. The authors reviewed promising computational tools and experimental platforms for interpreting noncoding SNPs. Which alter miRNA processing, stability, or target binding, necessitating the integrated multi-omic pipelines to link noncoding variation to phenotype (Wang and Hon, 2024).

Considering these studies demonstrate the variability and mechanistic importance of miR-eQTLs, isomiR-eQTLs, and regQTLs in influencing the cancer-based gene expression. In addition to seed binding, influence of miR-eQTLs is evident in miRNA biogenesis, isoform usage, or context-specific regulatory outcomes. As datasets grow and analytical methods improve, miR-eQTLs are poised to become central to our understanding of post-transcriptional regulatory variation in human cancer (Table 1).

**TABLE 1:** Summarizes major regQTL studies, resources, and databases that have systematically mapped miRNA-related regulatory variation across cancers and diverse populations.

Resource/Study	Description	Features/Scope	References
regQTL	SNP × miRNA interaction linear model	Allele-specific regulation in TCGA tumor types	Wilk and Braun (2018b)
miR-eQTL mapping	Genome-wide cis-miRNA QTL discovery	>5,200 cis-miR-eQTLs in blood; GWAS overlap	Huan et al. (2015)
isomiR-eQTL	Isoform-level miRNA QTLs across 30 cancer types	152k miR-eQTLs, 2.4M isomiR-eQTLs; GWAS overlap	Moradi et al. (2022)
Exposure-eQTL	Condition-specific regulatory SNPs in immune cells	Functional DNA damage context	Bigge et al. (2024)
TWAS for miRNA	miRNA expression association with prostate cancer	Model-trained miRNA expression risk predictors	Larson et al. (2022)
Asian miR-eQTL	Population-specific miR-eQTL discovery	Novel miRNAs in Asian ancestry cohorts	Sonehara et al. (2022)
miRNASNP v4	SNP annotation for miRNA and binding sites	Allele impact on binding and structure	Cao et al. (2025)
ENCORI	Experimentally supported miRNA–mRNA maps	CLIP-seq interaction evidence	Yang et al. (2011)
JAMIR-eQTL	Japanese-specific miR-eQTL database	Ethnic diversity inclusion	Akiyama et al. (2021)
ncRNA-eQTL Atlas	Multi-cancer noncoding RNA QTL mapping	miRNA, lncRNA eQTL annotations	Li et al. (2020)
MITHrIL	miRNA-aware pathway impact analysis	Enriched regulation prediction	Alaimo et al. (2016)

## 3 Computational strategies for regQTL and related miRNA regulatory analyses

Integrative and sophisticated computational methods are required for the study of regulatory quantitative trait loci (regQTLs), genetic variations that modify miRNA-mediated gene regulation. The traditional eQTL analyses link genetic variants to gene transcription levels. However, regQTL analyses are uniquely focused on how specific alleles modulate miRNA-mRNA functional interactions (Wilk and Braun, 2018b). This section reviews major computational frameworks and empirical studies that have advanced our understanding of regQTL biology in cancer and related contexts.

The importance of computational strategies in regQTL research cannot be overstated. Experimental studies alone are insufficient to capture the complexity of miRNA–mRNA regulatory networks, particularly given the context-dependent and allele-specific nature of these interactions. Computational models allow researchers to integrate genomic, transcriptomic, and epigenomic datasets to detect subtle regulatory effects that would otherwise remain obscured in bulk assays. These approaches also enable systematic screening of thousands of candidate variants, prioritization of functional SNPs for downstream validation, and the generation of mechanistic hypotheses that guide experimental design. As a result, computational methods form the essential foundation upon which experimental and translational regQTL discoveries are built.

### 3.1 Interaction regression models for allele-specific miRNA-mRNA regulation

**FIGURE 2:**
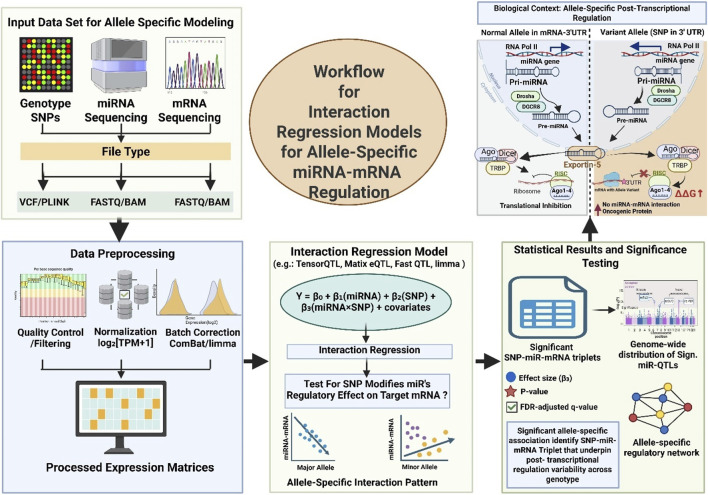
Workflow for Interaction Regression Models that Identify Allele-Specific miRNA–mRNA Regulation and its Biological Consequence. The data of genotype (VCF/PLINK), miRNA-seq, and mRNA-seq are integrated and further preprocessed (QC, normalization, and batch correction) to generate expression matrices. Next, a linear interaction regression model is applied to tests genotype-based modification are involve in miRNA regulation of target genes through the interaction term (β_3_). Further, significant SNP–miRNA–mRNA triplets help in interpreting the allele-specific regulatory effects that are visualized via Manhattan plots and network relationships. At end the biological interpretation are explored to see how a 3′UTR SNP can abolish miRNA–mRNA pairing, prevent RISC loading that further involve in overexpression of oncogenic protein.

The recent advancement in interaction-based modeling approaches let us understand about the influence of SNPs on miRNA-mRNA interaction (Wilk and Braun, 2018b). This computation model involves evaluating the genotype-based miRNA modulatory effects on its target. Thus providing cancer associated mechanistic insights into allele-specific post-transcriptional regulation where miRNA function is often disrupted (Jacinta-Fernandes et al., 2020). Recently Wilk and Braun introduced a linear interaction regression model that evaluates the SNP × miRNA interaction term. This model detects genotype-specific modulation of gene expression by miRNAs (Wilk and Braun, 2018b). Study included the transcriptomic and genotypic data from TCGA across multiple tumor types, including breast, lung, liver, and prostate cancers. This approach helps in identifying thousands of SNPs that significantly altered the miRNA–mRNA regulatory axis. It emphasizes the potential of regQTLs to explain inter-individual variability in cancer phenotypes. Moreover, it also allowed for the discovery of regulatory variants that were not evident through conventional eQTL mapping. Further expanding the scope of post-transcriptional genetic regulation in oncogenesis (Wilk and Braun, 2018b). The framework for identification of Allel-specific miRNA-mRNA regulation is illustrated in Figure 2.

In another study by Wilk and Braun further refined the methodology by incorporating biologically informed filters. They implemented the Isomap-based dimensionality reduction approach to identify miRNA–mRNA interaction pairs that were differentially regulated in tumors compared to normal tissue. This pathway-driven preselection of candidate regulatory relationships increased the statistical power and reduced false-positive findings in downstream regQTL modeling. Thus ensuring that only cancer-relevant miRNA–gene pairs were subjected to interaction testing (Wilk and Braun, 2018a). In another study, Yu et al. applied a similar interaction modeling approach to a multi-ethnic lung cancer cohort and replicated several cancer-specific regQTLs. They found that 3′UTR region of LAMC1 mRNA have polymorphism (rs3768617) that modulates the regulatory effect of miR-548b-3p. In addition genotype was significantly correlated with differential survival outcomes, highlighting its potential as prognostic biomarker (Yu et al., 2021).

Moving beyond proximal regulatory regions, recent study expanded the analytical landscape by incorporating 3D chromatin architecture into regQTL discovery. This approach employed Hi-C data and identified several SNPs through regQTL modeling. These regQTLs were found to located far from the miRNA or mRNA loci in enhancer–promoter loops. This spatial genomic context demonstrated that long-range chromatin interactions can significantly influence allele-specific regulation. This make complication in understanding the interplay between noncoding variation and gene expression regulation in cancer (Lu et al., 2020). Additionally, SNPs may not always have the same impact on miRNA–mRNA interactions under all biological contexts. To address this, Bigge et al. developed an innovative approach called exposure-dependent eQTLs (e^2^QTLs). They showed that SNP effects only become apparent in response to environmental stressors like genotoxic stress. In this study, the CD8^+^ T cells were exposed to DNA-damaging agents. Further, they identified e^2^QTLs that selectively influenced gene expression under stress conditions. Several influenced genes were associated with immune and apoptosis-related pathways. These findings suggest that some regQTLs may only manifest under specific tumor microenvironmental or therapeutic contexts, thus carrying implications for personalized oncology (Bigge et al., 2024).

Collectively, these studies establish a robust analytical foundation for the discovery and validation of regQTLs. The interaction regression model serves as a powerful and adaptable framework that enables researchers to probe allele-specific post-transcriptional regulation and uncover novel mechanisms through which noncoding variants contribute to tumorigenesis. Integrating additional layers such as pathway context, chromatin conformation, and environmental stimuli significantly enhances the interpretability and functional relevance of regQTLs. Which further advances in the study of cancer genetics and miRNA biology.

#### 3.1.1 Limitations and confounding in SNP × miRNA interaction models

Although SNP × miRNA interaction regression provides a principled framework for detecting allele-specific modulation of miRNA–mRNA relationships, these models are inherently susceptible to confounding. Technical batch effects, unobserved experimental variability, and latent biological structure can induce correlations between predictors and residuals that lead to inflated or entirely spurious interaction signals. Early foundational work by Leek and Storey demonstrated that hidden confounders in high-dimensional expression datasets generate “systematic, unmodeled covariance,” producing false-positive associations unless appropriate surrogate variables are included (Leek and Storey, 2007). Their findings directly apply to regQTL detection, because SNP × miRNA interaction terms are even more sensitive to unmodeled heterogeneity than standard eQTL effects.

Technical noise—including RNA extraction batch, sequencing run, library preparation kit, or RNA integrity—can introduce structured non-biological variation that distorts miRNA and mRNA abundance estimates. GTEx analyses showed that factors such as RNA integrity, ischemic time, sequencing platform, and sample batch substantially alter measured expression profiles and can bias both cis- and trans-eQTL estimates if not properly controlled (GTEx Consortium, 2017). When such variation correlates with genotype or miRNA abundance, SNP × miRNA interaction effects become uninterpretable.

Similarly, biological confounders such as population structure and tumor subtype can produce artifactual regulatory signals. Population stratification is a well-established source of spurious genotype–expression associations unless ancestry is explicitly modeled (Price et al., 2006). Moreover, miRNA expression varies substantially across tissues, cellular compositions, and tumor lineages, as demonstrated in large-scale cancer profiling studies (Lu et al., 2005). Biological heterogeneity is also known to distort QTL effect estimates, reinforcing the need to adjust for demographic, clinical, and ancestry-related covariates in interaction-based regQTL models (GTEx Consortium, 2017).

To address these sources of confounding, modern molecular QTL studies increasingly rely on latent-factor correction. Stegle et al. demonstrated that probabilistic factor models such as PEER effectively capture hidden determinants of gene expression arising from technical noise and uncontrolled heterogeneity, thereby improving both the power and reliability of molecular QTL inference (Stegle et al., 2012). Likewise, surrogate variable analysis (SVA) has been widely adopted for estimating unmeasured confounders and preventing false discoveries in high-dimensional genomic datasets (Leek and Storey, 2007). These methods are directly relevant to regQTL analysis and should be integrated into SNP × miRNA interaction frameworks.

The regQTL model proposed by Wilk and Braun acknowledged that miRNA–mRNA correlations are strongly influenced by tumor subtype and other clinical covariates, motivating the inclusion of relevant covariates in their interaction model (Wilk and Braun, 2018b). Although their implementation did not explicitly apply PEER or SVA, subsequent QTL studies have shown that latent-factor correction is essential for reducing false-positive interaction effects in large transcriptomic datasets.

Collectively, these findings underscore that SNP × miRNA interaction models cannot be interpreted without rigorous confounder control. Best practices include balanced experimental design, inclusion of technical and biological covariates, and application of latent-factor methods such as PEER or SVA. Without such corrections, regQTL signals may primarily reflect technical artifacts or latent structure rather than true allele-specific biological mechanisms.

### 3.2 Isoform-level analyses: mapping isomiR-eQTLs in cancer

**FIGURE 3:**
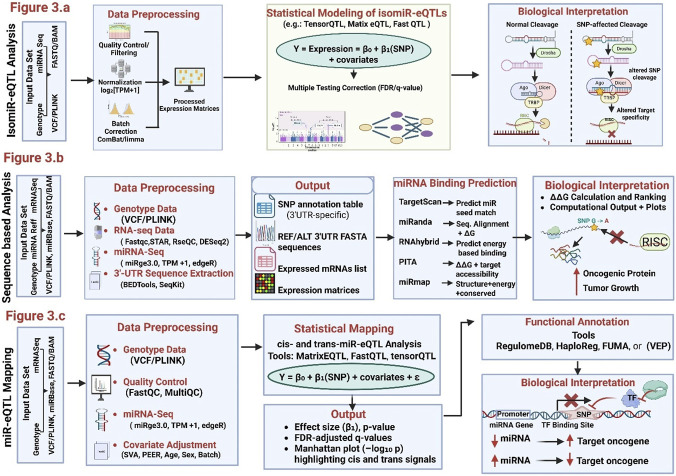
Integrated Computational and Biological Frameworks for miRNA QTL Analyses **(a)** IsomiR-eQTL Analysis: Workflow for identifying genetic variants that regulate isomiR expression patterns. Input genotype (VCF/PLINK) and isomiR-resolved miRNA-seq data undergo preprocessing (quality control, normalization log_2_ [TPM +1], and batch correction via ComBat/limma). Linear regression modeling (TensorQTL, MatrixeQTL, FastQTL) tests genotype–isomiR associations with FDR correction. Biological interpretation illustrates how SNP-altered DROSHA/DICER cleavage affects 5′/3′isomiR production and shifts miRNA target specificity. **(b)** Sequence-Based Binding-Disruption Analysis: Computational pipeline for predicting SNP-mediated disruption or creation of miRNA recognition elements (MREs) in 3′UTRs. Genotype, mRNA-seq, and miRNA-seq data are preprocessed to generate 3′UTR sequence sets and annotation tables. Binding-disruption predictions (TargetScan, miRanda, RNAhybrid, PITA, miRmap) compute ΔΔG and rank variants by allelic binding affinity. Biological interpretation depicts a G→A substitution disrupting miRNA–mRNA binding, impairing RISC loading, derepressing oncogenic mRNAs, and promoting tumor growth. **(c)** miR-eQTL Mapping: Computational and biological workflow for identifying SNPs that modulate miRNA transcription or processing. Genotype and miRNA-seq data are quality-controlled, normalized, and adjusted for covariates (SVA, PEER, age, sex, batch). cis- and trans-miR-eQTLs are mapped using MatrixEQTL, FastQTL, or tensorQTL. Functional annotation (RegulomeDB, HaploReg, FUMA, VEP) identifies regulatory variants within promoter or enhancer regions. Biological interpretation shows a promoter SNP disrupting transcription-factor binding, reducing pri-miRNA transcription and mature miRNA abundance, thereby derepressing oncogenic targets and driving tumor progression.

Although canonical miRNA sequences have been the main focus of traditional regQTL models. However, a growing body of evidence highlights the biological relevance of isomiRs-sequence. The isoforms of miRNAs arise from alternative cleavage, non-templated additions, or post-transcriptional modifications. These isomiRs can exhibit distinct expression patterns, seed sequences, and mRNA target profiles compared to their canonical counterparts. Consequently, the genetic regulation of isomiRs through isomiR expression QTLs (isomiR-eQTLs) has emerged as an important subfield in post-transcriptional regulation, especially in cancer (Moradi et al., 2022). A landmark study by Moradi et al. (2022) conducted the first comprehensive pan-cancer analysis of isomiR-eQTLs using data from 30 tumor types in The Cancer Genome Atlas (TCGA). The authors cataloged more than 2.4 million isomiR-eQTLs and demonstrated that many of these variants were located near known cancer-associated loci from genome-wide association studies (GWAS). Notably, over 90% of significant isomiR-eQTLs were distinct from canonical miR-eQTLs, highlighting the specificity of isoform-level regulation. This study also revealed tumor-type–specific isomiR–SNP associations, suggesting that isoform expression is finely tuned by both germline and somatic variation in a context-dependent manner (Moradi et al., 2022). The framework of isomiR-eQTLs mapping startagies are illustrated in Figure 3a.

In an earlier population-based study, Huan et al. (2015) performed genome-wide mapping of miR-eQTLs in whole blood from more than 5,000 individuals in the Framingham Heart Study. Their analysis identified over 5,200 cis-miR-eQTLs and provided one of the first systematic demonstrations that miRNA expression is under strong genetic control. While the focus was on canonical miRNAs, several of the identified eQTLs overlapped with isomiR-regulating regions identified in later cancer-focused studies, suggesting shared regulatory architecture across tissues and disease states (Huan et al., 2015). Beyond cancer, another study investigated isomiR regulation in healthy lymphoblastoid cell lines. Their findings suggest that isomiR expression is strongly influenced by both cis-acting SNPs and RNA-editing events. Their research offered strong evidence that 5′isomiR variations impacts the seed region that cause significant changes in target gene networks leading to increased SNPs based regulation (Ramachandran et al., 2016). Additionally, several other studies used high-throughput sequencing to quantify miRNAs at the isoform level in both normal and cancerous tissues. They highlighted the functional divergence of isomiRs in controlling different oncogenic pathways. Despite studies were explicitly not focus on eQTLs, it emphasized the need for isoform-aware pipelines in any analysis of genetic regulation involving miRNAs (Telonis et al., 2015; Tomasello et al., 2021; Moradi et al., 2022).

Together, these studies emphasize the necessity of distinguishing isomiRs from canonical miRNAs when mapping genetic regulatory effects. The identification of isomiR-eQTLs uncovers a hidden layer of post-transcriptional control, with implications for understanding cancer-specific gene regulation and identifying new functional variants missed by traditional eQTL analyses.

### 3.3 Sequence-based approaches: allele-specific miRNA binding site disruption

A critical mechanism by which SNPs influence miRNA-mediated regulation is by altering the binding affinity of miRNAs to their target sites, particularly within the 3′untranslated regions (3′UTRs) of mRNAs. These binding disruptions may result from gain or loss of miRNA recognition motifs and are typically modeled through computational prediction tools that evaluate thermodynamic binding energies or sequence complementarity. The eQTLs or regQTLs rely on expression-level correlations with the other factors. However, sequence-based models investigates modulatory effect of variations on the miRNA-mRNA interaction and direct biochemical implication (Rykova et al., 2022). Recent study evaluated a genome-wide screen of breast cancer susceptibility loci identified from GWAS. They determine association of SNPs with disruption or creation of novel miRNA-binding sites. In addition, an integrative pipeline having multiple prediction algorithms (TargetScan, miRanda, and RNAhybrid) was applied to calculate allele-specific differences in binding affinity (ΔΔG). These strategies revealed several SNPs that are involve in alteration of miRNA–target gene interactions. Moreover, this approach helps in identification of gene associated variants within the gene (e.g., CDH1, TOX3, and FGFR2). Several SNPs were located within linkage disequilibrium blocks of GWAS hits that reveals the potential mechanistic role in modulating breast cancer risk (Jacinta-Fernandes et al., 2020). The framework for sequence-based approach is illustrated in Figure 3b

Similarly recent database called PolymiRTS helps in cataloging the SNPs in putative miRNA seed-binding sites. This strategy identifies the polymorphisms disrupting conserved miRNA-binding motifs that were more likely to be associated with disease traits. Further results provide statistical support for the hypothesis that miRNA–target site disruption is a common disease mechanism. In addition, the prediction reliability is increased by incorporating conservation scores, expression evidence, and allele frequencies to rank functional impact (Ziebarth et al., 2012). Early study has also reported miRNA target site polymorphisms. Around 7% of common SNPs in 3′UTRs of target genes were potentially affect miRNA binding. These results provided the first population-scale estimate of regulatory variation affecting miRNA function and stimulated further development of computational tools to evaluate the functional impact of 3′UTR variants (Saunders et al., 2007). Similarly, another study introduced the concept of target site accessibility by incorporating mRNA secondary structure into binding predictions. The introduced PITA algorithm evaluated hybridization energy and the energetic cost of opening mRNA structures to make the binding site accessible. This added complexity has proven especially relevant when predicting subtle effects of SNPs that may not alter the seed sequence directly but still impair miRNA binding due to structural occlusion (Kertesz et al., 2007).

These studies have collectively established that sequence-level SNP effects on miRNA targeting are both prevalent and functionally impactful, especially in cancer. Such approaches are valuable for fine-mapping GWAS loci, identifying candidate causal variants, and guiding experimental validation of disrupted regulatory relationships.

#### 3.3.1 Modern sequence-based approaches for detecting allelic disruption of miRNA–mRNA interactions

Sequence-based prediction remains a foundational step in regQTL discovery, but early tools such as PolymiRTS and PITA capture only simple seed-pairing disruptions and lack the contextual features now known to shape miRNA–mRNA binding. Recent advances substantially improve methodological rigor by integrating evolutionary conservation, RNA structural accessibility, transcript isoform heterogeneity, and machine-learning–derived interaction scores.

TargetScan v8 is among the most widely used modern platforms for high-confidence identification of canonical miRNA recognition elements (MREs). Its updated algorithm incorporates refined seed-pairing rules, site context features, and improved 3′UTR alignments, providing markedly enhanced predictive precision for SNPs that disrupt or create functional miRNA-binding sites (McGeary et al., 2019). In parallel, miRDB uses the MirTarget machine-learning model, a support vector machine trained on thousands of experimentally validated miRNA-target interactions, enabling the systematic prediction of novel gene targets and the assignment of a confidence score (Chen and Wang, 2020). Both resources offer up-to-date and comprehensive coverage of human miRNAs. While they employ different computational approaches (TargetScan primarily uses conservation/context rules, miRDB uses a machine learning approach on experimental data), they are both considered indispensable, complementary tools in the field for downstream analysis like regulatory quantitative trait loci (regQTL) workflows.

Beyond canonical site prediction, specialized tools now evaluate allele-specific effects on miRNA binding with much greater biological realism. miRNASNP v3 integrates predictions of seed disruptions, creation of novel MREs, changes in minimum free energy, and effects on local RNA structure, enabling fine-resolution identification of functional 3′UTR variants (Liu et al., 2021). Deep-learning models such as miRAW and miRBind capture nonlinear features of miRNA–mRNA pairing—including bulges, mismatches, and noncanonical interactions—significantly outperforming earlier heuristic tools by leveraging large experimental training datasets (Pla et al., 2018; Klimentová et al., 2022).

Notably, newer frameworks increasingly integrate CLIP/eCLIP binding maps and RNA structural profiling, enabling prediction of sequence variants that alter Argonaute or RBP occupancy—mechanisms highly relevant for regQTL discovery. This shift reflects a broader field-wide recognition that miRNA targeting is shaped not only by seed complementarity but also by local RNA architecture, isoform-specific 3′UTR usage, and competitive interactions with RNA-binding proteins (Van Nostrand et al., 2020).

Together, these modern sequence-based tools provide a significantly more accurate and biologically informed foundation for identifying candidate regQTLs. Their integration into computational pipelines is now essential for reducing false-positive predictions and ensuring methodological relevance, directly addressing the reviewer’s concern regarding reliance on outdated resources.

### 3.4 miR-eQTLs and population-level genetic regulation of miRNA expression

While much of the regQTL literature emphasizes SNPs that influence miRNA–mRNA interactions. Several recent studies have explored how genetic variation directly affects miRNA expression itself. These miRNA expression quantitative trait loci (miR-eQTLs) represent a foundational layer of miRNA regulation. The variation in regulatory regions (e.g., promoters, enhancers, and processing elements) alter the abundance of mature miRNAs across individuals. Such insights are critical for understanding the heritable component of miRNA expression variation, especially in disease contexts such as autoimmune disease, *etc.* (Gamazon et al., 2013; Wohlers et al., 2018; Villegas-Mirón et al., 2022). A recent population-based study by Huan et al. (2015) mapped 5,269 cis-miR-eQTLs for 76 miRNAs in blood from 5,239 individuals. Study revealed many of the regulatory variants lie 300–500 kb upstream of miRNA loci. These miR-eQTLs overlapped significantly with mRNA-eQTLs and GWAS hits including rs7115089 affecting miR-125b-5p and HDL cholesterol levels. The study underscored the role of miRNAs as genetic mediators of complex traits (Huan et al., 2015). The framework for miR-eQTLs and population level genetic regulation is illustrated in Figure 3c.

Similarly, Rotival et al. (2020) analyzed 977 miRNA-seq profiles from monocytes of African and European individuals under immune stimulation. While activation altered miRNA and isomiR expression, changes were modest compared to mRNAs, suggesting tighter evolutionary constraint. They identified context-independent miR-QTLs and found that population-level miRNA differences were mainly non-genetic, though ∼60% could be explained by miR-QTLs where present. An adaptive expansion of a miRNA cluster on chromosome 14 was noted in Europeans. Overall, miRNA–mRNA correlations were driven more by co-transcription than by transcript degradation (Rotival et al., 2020).

Recent meta-analysis assessed the polymorphisms in core miRNA biogenesis genes such as XPO5 and RAN. These genes are responsible for nuclear export and transport of miRNA precursors to the cytoplasm. The study employed Bayesian hierarchical modeling of multiple cancer GWAS datasets. Their finding suggests that specific variants such as XPO5 (rs11077) and RAN (rs3803012) were consistently associated with increased cancer susceptibility. These findings suggest that polymorphisms present in these genes significantly alter global miRNA maturation efficiency. Further, indirectly affecting downstream miRNA abundance and regulatory activity (Shao et al., 2020). Similarly, several studies explored the role of miRNA-promoter based SNPs that impact transcriptional regulation. Findings suggest that regulatory SNPs upstream pre-miR-30c-1, miR-146a and miR-34b/c disrupts transcription factor binding motifs that lead to altered expression in cancer cell lines. These observations suggest that promoter-localized genetic variation may serve as an upstream control point for miRNA-based gene regulation (Chou et al., 2010; Hu et al., 2011; Luo et al., 2011).

Collectively, these population-level studies demonstrate that miRNA expression is under strong genetic control and that both cis-acting SNPs and variants in the miRNA biogenesis machinery can significantly contribute to inter-individual and ancestry-specific differences in miRNA profiles. This layer of regulation adds further complexity to interpreting disease associations, as it implicates not only miRNA-target interactions but also miRNA dosage as a heritable trait. As this is a growing field, hence we have summarized the computational tools and resources used in regQTL and related miRNA regulatory analyses (Table 2).

**TABLE 2:** Computational tools and resources for regQTL and related miRNA regulatory analyses.

Tool/Method	Type	Data input	Cancer type applied	Key references
miRNASNP v3.0	Functional annotation database	SNP, miRNA, and target prediction	General/cancer	Liu et al. (2021)
SMR/HEIDI	Summary-data-based Mendelian Randomization	GWAS and eQTL summary data	Colorectal, breast	Zhu et al. (2016), Jayarathna et al. (2021)
miR-QTL Mapping	Genome-wide miR-eQTL discovery	Genotype and circulating miRNA expression	Population-level (plasma)	Huan et al. (2015)
QTLtools	QTL mapping framework	Genotype and expression matrices	Multi-tissue/cancer (GTEx/TCGA)	Delaneau et al. (2017)
sQTLseekeR	Splicing QTL discovery	Genotype and RNA-seq splicing data	TCGA	Monlong et al. (2014)
Matrix eQTL	Fast eQTL association testing	Genotype and expression matrix	Widely used in cancer	Shabalin (2012)
PEER	Confounder correction tool	Expression data	Pan-cancer/GTEx	Stegle et al. (2012)
GTEx Portal Tools	miR-eQTL and tissue-wide QTLs	Expression, miRNA, genotype data	Multi-tissue (normal, cancer)	The GTEx Consortium (2020)

### 3.5 miRNA-TWAS as a computational prioritization tool for regQTL analysis

Recent extensions of transcriptome-wide association studies (TWAS) to microRNAs have shown that genetically regulated miRNA expression can be associated with cancer susceptibility. In prostate cancer, a miRNA-TWAS identified significant associations for miR-941 and miR-3617-5p, demonstrating the feasibility of genetically predicted miRNA expression models (Larson et al., 2022). Similarly, a colorectal cancer miRNA-TWAS highlighted miR-1307-5p and miR-192-3p as susceptibility miRNAs whose genetically predicted expression was associated with disease risk (Chen et al., 2024). These efforts build on foundational work showing that many mature miRNAs exhibit strong cis-genetic regulation, enabling reliable genetic prediction models (Huan et al., 2015).

Although no published study has yet used miRNA-TWAS to directly discover regQTLs, it provides a valuable upstream computational filter for regQTL studies. TWAS highlights miRNAs whose expression is both genetically regulated and phenotype-associated, allowing downstream regQTL scans to focus SNP × miRNA × mRNA interaction testing on a smaller, biologically informed subset. These complements established regQTL frameworks that directly test interaction effects between genotype, miRNA expression, and mRNA abundance (Wilk and Braun, 2018b; Yu et al., 2021). Integrating TWAS-based miRNA prioritization with interaction-based regQTL modeling represents a promising direction for future computational studies of germline-modulated miRNA–mRNA regulation.

## 4 Biological validation and functional relevance of miRNA regulatory QTLs in cancer

Building on computational regQTL discovery, the SNPs modulatory effect on miRNA-mRNA at biological and clinical level is essential. These regulatory variants influence post-transcriptional gene expression by altering miRNA binding, processing, or stability. This mechanism ultimately affecting oncogenic signaling, immune modulation, and therapy response. This section outlines functional validation studies, clinical associations, and emerging mechanistic frameworks that connect regQTLs to tumor biology through experimental and multi-omics evidence.

### 4.1 Functional validation of candidate regQTLs

A compelling demonstration of regQTL function emerges from Jacinta-Fernandes et al. (2020), who experimentally confirmed that the SNP rs4245739 in the *MDM4* 3′UTR disrupts miR-191 binding. Luciferase reporter assays in breast cancer lines revealed allele-specific repression, strongly supporting that computationally predicted variants can meaningfully influence tumor suppressor regulation (Jacinta-Fernandes et al., 2020). Similarly, Kjersem et al. (2012) revealed the SNPs (rs61764370) in the *KRAS* 3′UTR region that prevents the binding of miR-Let7 has direct effect on overexpression of *KRAS* gene in colorectal cancer (Kjersem et al., 2012). In lung cancer, rs3768617 in LAMC1 was identified as a functional regQTL-SNP: the variant T allele disrupted miRNA-548b-3p–mediated repression, elevated LAMC1 expression, and promoted tumor growth. Functional assays, including LAMC1 knockdown, confirmed its oncogenic role, providing one of the first *in vitro* and *in vivo* validations of a candidate regQTL in lung cancer and suggesting that precision approaches, such as CRISPR/Cas9 editing of regQTL variants, could open new avenues for targeted intervention (Yu et al., 2021). A broader overview by Hajibabaie et al. (2022) reviewed fifteen empirically confirmed miR–SNP interactions in gastrointestinal cancers, verified using luciferase reporters or allele-specific expression assays. Together, these studies highlight the reliability of regQTL models when combined with targeted functional testing in meaningful cancer systems (Hajibabaie et al., 2022). A summary of validated miRNA-targeting SNPs has shown in Table 3.

**TABLE 3:** Summary of functionally validated miRNA-targeting SNPs (regQTLs) across cancer types.

SNP (gene)	Affected miRNA	Cancer type	Functional assay	Observed functional impact	Key references
rs4245739 (MDM4)	miR-191	Breast	Dual-luciferase reporter	Variant allele abolishes miRNA-mediated repression	Wynendaele et al. (2010), Stegeman et al. (2015), Jacinta-Fernandes et al. (2020)
rs61764370 (KRAS)	miR-let-7	Colorectal	Reporter binding assay	Loss of miR-216b repression - upregulated KRAS	Chin et al. (2008), Zhang et al. (2011), Saridaki et al. (2014)
rs3768617 (LAMC1)	miR-548b-3p	Lung adenocarcinoma	CRISPR-edited cell assays	Altered repression and increased invasiveness	Yu et al. (2021)
rs2910164 (pri-miR-146a)	miR-146a	Head and Neck (mouse)	*In vivo* vaccine model	Genotype-specific immune priming	Liu et al. (2022)
rs767649 (DC cells)	miR-155	Dendritic cell assay	Cytokine functional assay	IL-12 secretion altered; restored with mimic	Iranparast et al. (2023)
rs1815009/rs2684788 (IGF1R)	miR-133a/b, miR-455	Prostate	Dual-luciferase assay	Allele-specific miRNA suppression differences	Wang et al. (2019)
rs8176318 (BRCA1)	miR-525-5p	Breast/Colon	Reporter and epidemiology	Genotypes linked to altered miRNA binding risk	AHMAD et al. (2019)
rs10878441 (LRRK2)	miR-?	Breast	Genotyping + survival analysis	CC genotype associated with poorer prognosis	Zhang et al. (2021)
rs16917496 (SET8/KMT5A)	miR-502	Breast	TaqMan + survival mapping	TT genotype significantly associated with poor survival	Song et al. (2009)
miRSNPs (GREM1, ITGB4)	Various	Breast/other	Cohort association studies	SNPs associated with subtype-specific prognosis	Zhang et al. (2021)
Various SNPs in several genes	Various	Gastric and Colorectal Cancers	Meta-analysis + functional assays	SNP-miRNA interactions linked to prognosis and invasion	Hajibabaie et al. (2022)
SNPs in ABHD8	miR-4707-3p	Breast	Reporter assay	Altered binding, affects protein level	Li et al. (2017), Jacinta-Fernandes et al. (2020)
BRCA1/2, RAD51C/D, SLFN11, etc.	MiR-21 and let-7	Ovarian	Pharmaco-genomic profiling	SNPs predict PARP inhibitor sensitivity	Bai et al. (2022)

### 4.2 Connecting regQTLs to clinical phenotypes

A growing body of evidence connects regulatory SNPs that alter miRNA-mRNA binding (regQTLs/miR-SNPs) to patient phenotypes disease risk, survival, and treatment responses supporting their translational potential as biomarkers. A well-characterized example is rs3768617 in *LAMC1*: the T allele weakens miR-548b-3p–mediated repression, elevates *LAMC1* expression, and promotes malignant phenotypes; *LAMC1* knockdown reduces tumor growth *in vitro* and *in vivo*. Although primarily reported as a risk-associated regQTL for lung cancer rather than a survival marker, this study provides rigorous functional validation across molecular and phenotypic layers (Yu et al., 2021). Multiple clinically relevant regQTLs have been reported across cancer types. SET8 (KMT5A) rs16917496 (miR-502 site) has been linked to clinical outcomes in multiple cohorts; in NSCLC, rs16917496 (T>C) is associated with survival via altered miR-502 regulation of SET8 (Xu et al., 2013; DOI: 10.1371/journal.pone.0077024), and consistent signals have also been reported for SCLC outcomes and ovarian cancer risk in related studies on the same binding site (Xu et al., 2013). Similarly, MDM4 rs4245739 (creates a miR-191 site) reduces MDM4 expression and has been associated with cancer susceptibility and, in some reports, clinical outcomes; pooled analyses/meta-analyses support its clinical relevance, with disease-specific nuances such as esophageal squamous cell carcinoma (Zhou et al., 2013). Another important category involves the immune checkpoint axis, where PD-L1 3′UTR miR-sites (e.g., rs4143815, rs2297136) have been repeatedly linked to tumor PD-L1 expression and prognosis across cancers, including NSCLC, with several analyses implicating miR-570 binding at rs4143815 (Lee et al., 2016; Ohhara et al., 2024). Additionally, a CD133/Prom1 miR-135a/b site variant has been associated with favorable prognosis in lung cancer, consistent with reduced oncogenic potential via strengthened miRNA repression (Du et al., 2017).

In addition to prognostic significance, several miRNA-binding-site polymorphisms act as predictive biomarkers for therapy response. Anti-EGFR therapy: The KRAS let-7 site variant (rs61764370) - a classic miR-binding-site polymorphism has shown associations with treatment response in subsets of metastatic colorectal cancer (mCRC) patients receiving anti-EGFR antibodies, and survival in certain head-and-neck/oral cancer cohorts, though findings are disease- and study-specific and sometimes contested (Cerne et al., 2012). Similarly, cytotoxic chemotherapy: miRNA-binding-site SNPs in ETS2 predicted paclitaxel–cisplatin outcomes in advanced NSCLC, highlighting how regQTLs can stratify chemotherapy benefit (Hong et al., 2016). Immune checkpoint inhibitors (ICIs): *PD-L1* 3′UTR variants (e.g., rs4143815, rs2297136) have been associated with ICI response and survival in NSCLC cohorts treated with nivolumab/pembrolizumab—an emerging line of evidence that germline variation in miRNA-binding sites can shape immunotherapy outcomes (Yoshida et al., 2021). Collectively, these studies demonstrate that regQTLs can (i) shift gene expression by altering miRNA repression, (ii) map to risk or prognosis in tumor- and ancestry-specific ways, and (iii) act as predictive markers for targeted therapy, cytotoxic chemotherapy, and ICIs. Notably, some associations (e.g., KRAS rs61764370) show context-dependence or conflicting reports, underscoring the need for replication in large, well-phenotyped cohorts, ancestry-aware modeling, and functional validation in relevant tumor contexts before clinical adoption (Weidhaas and Slack, 2011).

### 4.3 Mechanistic dissection via multi-omics and single-cell studies

Advancement of field suggests that bulk expression data and standard reporter assays are not sufficient to confirm allele-specific miRNA regulation. Resolution of this issue totally relies on high-resolution experimental approaches that can capture regulatory events directly within their native cellular environment. These experimental tools allow the identification of mechanisms behind regulatory role of regQTLs at tissues, individual cell types, and even the surrounding microenvironment level. One example is the use of crosslinking immunoprecipitation followed by sequencing (CLIP-seq). This method does not dependent on the artificial reporter system instead they directly capture miRNA–mRNA interactions inside the cell. This technique tests that predicted SNP have ability to change interaction between Argonaute-bound miRNAs-target transcripts. Such direct evidence strengthens the causal influence that cannot predicted with computational methodology alone (Chi et al., 2009). Such methodologies provide a gold standard for validating regQTL function in tumor tissue.

The mapping of regulatory variation has been greatly improved after integrating single-cell RNA sequencing (scRNA-seq) with genotype information. This integration helps in identifying pinpoint variants effect withing cell types. The recent study by van der Wijst et al. analyzed 25,000 PBMCs from multiple donors. They identified numerous cis-eQTLs that were restricted to specific immune cell subtypes. Most importantly these cis-eQTLs were not detected in bulk transcriptome data. These results revealed regulatory SNPs also have cell-type specific effects that are masked in bulk transcriptome (van der Wijst et al., 2018). Developing studies also showed that regQTL effects can be highly context dependent. In many cases, these regulatory impacts appear only under specific environmental conditions, such as DNA damage or inflammatory signaling. Recent study by Bigge et al. examined CD8^+^ T cells from healthy donors after exposure to a panel of DNA-damaging agents. This exposure created a stress in cells that further activates set of regulatory variants of exposure eQTLs (e^2^QTLs) that effects apoptotic gene expression. This type of dynamic regulation explain the effect of regQTLs on treatment response rather than baseline gene expression (Bigge et al., 2024).

All these studies multi-omics and single-cell studies establish that regQTLs operate within a layered biological framework. Which includes 1) Molecular assays (e.g., CLIP-seq) that confirm altered miRNA binding *in vivo*, 2) Single-cell approaches that uncover cell-type specificity in tumors 3) Context-sensitive eQTL analyses that reveal conditional effects under therapy or stress. This integrative view shows that regQTLs are not static modifiers of gene expression but dynamic regulators whose impact depends on tissue architecture and microenvironmental cues. Such insights are vital for translating regQTL discoveries into precision oncology applications.

#### 4.3.1 Systematic strategies for functional validation of regQTLs

Although computational and multi-omic evidence suggests that regQTLs may influence miRNA–mRNA interactions and post-transcriptional regulation, systematic functional validation remains largely unexplored. Below are robust experimental and genomic strategies—validated in related contexts—that could realistically be repurposed to test regQTL hypotheses at scale.

- Massively Parallel Reporter Assays (MPRAs): MPRAs allow high-throughput functional screening of thousands of allele-specific effects on transcript regulation. This approach has successfully identified hundreds of expression-modulating noncoding variants across human cell types (Tewhey et al., 2016). Recent meta-analyses and methodological reviews highlight MPRAs as a scalable method for testing variant functions in cis-regulatory DNA and RNA elements (La Fleur et al., 2024). A rigorous MPRA screening of candidate regQTLs (e.g., 3′UTR variants predicted to alter miRNA binding) would significantly reduce the number of false positives and narrow the focus to variants with measurable regulatory effects.
- CRISPR-based perturbation and miRNA-perturbation screens: CRISPR/Cas9 editing or CRISPR interference/activation (CRISPRi/a) can be used to introduce or correct regQTL alleles in the genome and assess their effect on gene expression, miRNA binding, and cellular phenotypes. Although widely applied to coding and noncoding regulatory regions, CRISPR-based functional screens targeting miRNAs or noncoding variants remain underutilized. A recent study demonstrated feasibility by generating a miRNA-targeting CRISPR-Cas9 knockout library in multiple cancer cell lines, revealing essential miRNAs for cell fitness (Merk et al., 2024). This establishes a robust platform that could be extended to allele-specific regQTL validation in relevant cancer models.
- Integrative multi-omic assays and single-cell perturbation: Crosslinking immunoprecipitation methods (e.g., eCLIP) enable *in vivo* measurement of RNA–protein interactions and can detect allele-specific differences in miRNA–mRNA binding and RISC recruitment. For instance, allele-biased eCLIP has been used to map how genetic variants influence RNA binding protein associations across the transcriptome (Gallego Romero and Lea, 2023). In parallel, single-cell CRISPR perturbation platforms (e.g., Perturb-seq) can dissect how regQTL alleles affect target gene expression and cellular state in heterogeneous tumor or tissue samples. While direct application to regQTLs remains to be demonstrated, this combination of genome editing, single-cell transcriptomics, and allele-aware binding assays represents a powerful, biologically relevant validation framework.

### 4.4 Mitigating bias and modeling population context

The translation values of regQTLs are very high, however, methodological and biological prejudices make difficulties in interpretation. Moreover, differences in tumor purity and the number of stromal cells within the sample are also alarming concerns. In addition, use of relative bulk tumor transcriptomes, and the abundance of malignant, stromal and immune cells also skews the regulatory association. In recent studies integration of tumor purity led to withdrawal of many putative eQTLs. The inclusion of purity estimation in models enhances the detection of variants with higher accuracy and specificity (Geeleher et al., 2018). These findings emphasize the consideration of tissue composition during regQTL analysis. Moreover, the optimal resolution of cell type specific regulatory effects requires incorporation of single cell data or validated computational deconvolution framework. Similarly, population diversity creates additional layer of complexity. The differences in allele frequencies and linkage disequilibrium pattern differ in population resulting in different genetic regulation of miRNA-mRNA interactions. Recent study on larger populations provided strong evidence of this phenomenon. A UK biobank based phenome-wide study revealed several miRNA-related variants with both regulatory and disease association (Mustafa et al., 2023). In a follow-up study, they mapped plasma miR-eQTLs in the Rotterdam Study and linked genetic effects on circulating miRNAs to a range of clinical traits (Mustafa et al., 2024). Together, these findings highlight the need for multi-ethnic datasets to ensure regQTL biomarkers are broadly applicable.

In addition, emerging evidence suggests the potential role of circulating miRNAs as non-invasive regQTL biomarkers. The non-invasive nature of these regQTLs biomarkers poses a realistic prospect to relate genetic variability with regulatory effects. A recent study involving Framingham Heart Study has mapped miRNA eQTLs derived from plasma of 3,743 patients. Their analysis revealed strong and reproducible genetic effects on circulating miRNA levels (Huan et al., 2024). These findings reveal that germline variation is involved in shaping the tumor-intrinsic gene expression and diversity of circulating miRNA. Moreover, findings are highly relevant for liquid biopsy-based stratification of patients. However, the association of plasma derived miRNA and tumor-derived miRNA regulation need to be carefully evaluated.

Collectively, minimizing bias in regQTL studies requires careful adjustment for tumor purity and stromal admixture. It also requires the use of ancestry-aware statistical models supported by diverse and multi-ethnic cohorts. Finally, circulating and tumor-intrinsic regulatory effects must be interpreted cautiously else they may reflect distinct biological contexts. Addressing these challenges is essential for moving regQTLs beyond computational discovery. Only then can they mature into reliable biomarkers with real clinical utility.

## 5 Translational and therapeutic implications of miRNA-associated regQTLs in cancer

As the landscape of miRNA regulatory quantitative trait loci (regQTLs) matures, their discovery offers new frontiers in precision oncology-from predictive diagnostics to innovative therapeutic strategies. In this section we discussed how regQTLs are translating into clinical potential, enriched by studies from the last 10 years.

### 5.1 regQTLs as biomarkers for drug response and treatment personalization

The identification of regQTLs that disrupt or enhance miRNA-mRNA interactions has opened new avenues for understanding inter-individual variation in drug response. These variations in SNPs cause dysregulated post-transcriptional gene regulation and can be used as biomarker to assess chemotherapy sensitivity, resistance and toxicity. Early large-scale computational efforts have laid the foundation for this new avenue of research. Recent study has systematically mapped regQTLs in several tumor types. They revealed that germline variants can significantly alter miRNA-target interactions and contribute towards variation in therapeutic outcomes (Wilk and Braun, 2018b). While this study was exploratory, it underscored the translational value of incorporating regulatory variants into biomarker discovery pipelines.

Several patient-based investigations have begun to establish clinical relevance. In recent study revealed that CRC patients receiving capecitabine-based treatment carry specific pre-miRNA polymorphisms. These polymorphism (*rs744591* and *rs745666*) were found to associated with improved response rates and differential toxicity profiles (Mao et al., 2017). Similarly, in non-small cell lung cancer patients treated with platinum-based chemotherapy have shown the modulatory effect of polymorphisms such as *rs11614913* (miR-196a-2) and *rs928508* (miR-30c-1). This highlights the predictive capacity of miRNA-related SNPs in guiding therapeutic decisions (Fang et al., 2018). Similarly, broader evidence of clinical relevance of miRNA-associated variants was explored in systemic evaluation. Recent study by Hajibabaie et al. (2022) explored the 15 independent studies and identified 20 distinct SNPs located withing the miRNA binding or precursor regions. These SNPs were highly associated with prognosis, treatment response and survival outcomes in gastric and CRC malignancy (Hajibabaie et al., 2022).

Collectively, these findings reinforce the notion that regQTLs may act as clinically useful biomarkers, particularly in drug classes where gene expression regulation plays a decisive role, such as DNA repair or drug metabolism pathways. Despite these advances, most current associations remain correlative, and direct functional validation of allele-specific miRNA binding and mechanistic impact on drug sensitivity is limited. Future research should integrate genotyping with functional assays (e.g., luciferase reporter systems, CLIP-seq) and well-powered clinical studies to confirm causality. Such efforts will be critical to translating regQTL discoveries into actionable biomarkers for precision oncology.

### 5.2 Liquid biopsy and circulating miRNA-eQTLs: broadening the translational horizon

Liquid biopsies-based profiling of circulating miRNAs offers less invasive insight to assess the tumor biology and systemic regulation. The mapping of expression quantitative trait loci for circulating miRNAs (miR-eQTLs) has expanded the clinical potential of regQTLs beyond tissue-specific contexts, suggesting that germline variants may shape not only tumor-intrinsic gene regulation but also the extracellular miRNA milieu accessible through blood samples. The recent studies based on genome-wide analyses revealed the potential of miRNA-eQTLs as liquid biopsy base biomarker. In the Rotterdam Study on 2,178 participants has identified 3,292 significant association that links 1,278 germline SNPs with 63 plasma derived miRNA (Mustafa et al., 2024). Most importantly, around 65% of miR-eQTLs are reproducible in independent cohorts. Much of these variations were mapped to the same genomics region. Together, these results suggest that germline genetic variation influences the circulating miRNA levels that further intersect with cancer associated pathways. Another study identified more than 5,000 plasma derived circulating miR-eQTLs that are heritable and overlapping nature with cancer associated loci (Huan et al., 2015).

In addition, circulating miRNAs are shown to have diagnostic and predictive utility for clinical settings. In recent study, around 25 exosomal miRNAs were evaluated in patients with lung adenocarcinoma. Out of 25 the eight-miRNA panel (including miR-21-5p, miR-126-3p, miR-210-3p, and let-7b-5p) was found to have higher correlation with histology and clinicopathological features (Hassanin and Ramos, 2024). Similarly, differential expression of several circulating miRNAs was evaluated for staging biomarker in early breast cancer patients with sentinel lymph node metastases (Escuin et al., 2021). Moreover, many of these miRNAs were regulated by germline variation. Further, integration of the plasma-based miRNA level with genotype could help in risk stratification. Tumor-derived exosomal miRNAs also appear to contribute directly to disease progression. Bayat and Sadri Nahand (2024) describe how exosomal miRNAs can remodel distant tissue microenvironments, priming pre-metastatic niches through immune and stromal modulation. Similarly, Guo et al. (2019) summarize mechanisms such as extracellular vesicle-mediated inflammation, matrix remodeling, and immune suppression that help establish metastatic sites before tumor cell arrival. If components of these exosomal cargoes are genetically regulated by regQTLs, then germline variation could influence metastatic potential and systemic disease behavior.

Collectively, these findings position circulating miRNA regQTLs as powerful, minimally invasive biomarkers with applications in cancer diagnosis, staging, therapy monitoring, and potentially metastasis prediction (Table 4). However, rigorous validation is required to disentangle tumor-derived *versus* host-derived contributions and to translate these associations into clinically actionable liquid biopsy assays.

**TABLE 4:** Experimentally validated regQTLs in cancer: functional assays and clinical associations.

Gene/Target	SNP ID	Affected miRNA	Cancer type	Functional validation	Clinical association	Key studies
MDM4	rs4245739	miR-191	Breast, ESCC, others	Luciferase reporter, allele-specific repression	Cancer susceptibility, prognosis	Zhou et al. (2013), Hong et al. (2016)
KRAS	rs61764370	let-7	Colorectal, H&N, oral	Disruption of let-7 binding, oncogene overexpression	Predictive for anti-EGFR therapy; survival impact (context-specific)	Weidhaas and Slack (2011), Kjersem et al. (2012)
LAMC1	rs3768617	miR-548b-3p	Lung	Reporter assays, knockdown and xenograft validation	Risk allele promotes tumor growth	Yu et al. (2021)
SET8 (KMT5A)	rs16917496	miR-502	NSCLC, ovarian	Reporter assays, allele-specific repression	Prognostic marker (overall survival, relapse-free survival)	Xu et al. (2013)
PD-L1	rs4143815, rs2297136	miR-570, others	NSCLC, gastric	Allele-specific regulation of PD-L1 3′UTR	Prognosis, ICI therapy response	Lee et al. (2016), Nomizo et al. (2017), Ohhara et al. (2024)
CD133/PROM1	variant in miR-135a/b site	miR-135a/b	Lung	Functional allele-specific repression assays	Favorable prognosis	Cheng et al. (2013)
ETS2	SNP in 3′UTR	miRNA binding site	NSCLC	Reporter assays	Predictive of paclitaxel–cisplatin chemotherapy response	Hong et al. (2016)

### 5.3 Therapeutic targeting of allele-specific miRNA regulation

Therapeutic strategies that modulate miRNA pathways provide several routes to counteract the consequences of regQTLs that weaken or strengthen miRNA–mRNA binding. In the clinic, three proof-of-concept modalities have reached human testing: (i) miRNA mimics (to restore tumor-suppressive miRNAs), (ii) anti-miRs/antagomirs (to inhibit oncomiRs), and (iii) target-site blocking or editing approaches that act directly at the miRNA response element (MRE). The first-in-human, multi-center phase I trial of the liposomal miR-34a mimic (MRX34) in advanced solid tumors demonstrated pharmacodynamic target modulation and partial responses but was halted early due to immune-mediated toxicities, underscoring both feasibility and current safety challenges for systemic miRNA replacement (Hong et al., 2020). Related experience with miR-16 mimic “TargomiRs” (EM-E6B-CU, MesomiR-1) delivered via EGFR-targeted bacterial minicells in recurrent malignant pleural mesothelioma reported acceptable safety and disease control in a phase I setting, highlighting targeted delivery as a key determinant of tolerability (van Zandwijk et al., 2017).

Outside oncology but highly informative for drug class feasibility, the anti-miR miravirsen (LNA inhibitor of miR-122) achieved dose-dependent, prolonged suppression of HCV viremia in a randomized trial—an important precedent for durable miRNA inhibition in humans (Janssen et al., 2013). In hematologic malignancies, cobomarsen (MRG-106), an LNA inhibitor of miR-155, showed mechanism-consistent pathway modulation preclinically and entered early-phase testing for CTCL, further supporting clinical tractability of anti-miRs (Seto et al., 2018). Because many regQTLs act by altering a single MRE on a specific transcript, site-directed strategies are particularly attractive. “Target-site blockers/target protectors” are short oligonucleotides designed to mask an MRE in one mRNA without globally inhibiting the cognate miRNA; they provide allele- and transcript-level precision and have been validated in cultured cells and vertebrate models (Knauss et al., 2013; Lima et al., 2018). Complementing pharmacologic masking, genome editing can now install or correct single-nucleotide changes in the endogenous 3′-UTR to test or repair MRE function. A TALEN-based *in vivo* study in zebrafish ablated a miR-430 site to prove causal regulation by a native MRE (Nat Commun, 10.1038/ncomms5640). In human cells, a CRISPR/HDR framework (following an MPRA-based screen) systematically edited 3′-UTR variants and validated causal alleles including one that disrupts a miRNA site in TRIM14 directly linking genotype to expression change (Griesemer et al., 2021). Most recently, prime editing was used to precisely install functional 3′-UTR variants in cancer-relevant genes (MFN2, FOSL2, IRAK1), demonstrating a route to allele-level correction when regQTLs rewire post-transcriptional control (Fu et al., 2024).

High-throughput genetics further informs which miRNAs or MREs are therapeutically actionable. Early CRISPR screens targeting miRNA genes in leukemia cells showed that specific miRNAs can be fitness-essential, nominating them and their target networks for therapeutic modulation (Wallace et al., 2016). Conceptually, integrating such screen outputs with germline regQTL maps can prioritize SNP–miRNA-mRNA triplets were correcting a single allele (or masking one MRE) is most likely to yield therapeutic benefit. Finally, delivery technology remains the rate-limiting step for miRNA drugs aimed at allele-specific regulation in solid tumors. Lessons from the broader RNA-therapeutics field (lipid nanoparticles, ligand-targeted carriers, and chemical stabilization) now offer rational design rules to improve tumor uptake while limiting immune activation-an advance that will be crucial for translating allele-targeted mimics, anti-miRs, and MRE-directed oligonucleotides (Paunovska et al., 2022; Martino et al., 2025).

### 5.4 Integration of deep learning, pharmacogenomics, and miRNA-regulatory networks

As cancer biology grows in complexity, integrating regQTL data with machine learning and pharmacogenomic frameworks is becoming an important frontier for precision oncology. These approaches allow the functional consequences of allele-specific miRNA regulation to be modeled alongside broader molecular features, providing predictive insights into drug sensitivity, immunotherapy response, and opportunities for drug repositioning. Early proof-of-concept work demonstrated the potential of neural networks to capture genotype–phenotype associations. Kalinin et al. (2018) developed a deep learning framework combining mutation and gene-expression data to predict drug sensitivity across cancer cell lines. While not explicitly incorporating miRNA regulation, the design provides a prototype for embedding regQTL features into pharmacogenomic prediction models. Chatzopoulou et al. (2022) extended this idea by building pipelines that integrated miRNA–mRNA networks with drug-response datasets. Their finding revealed disturbance in regulatory architecture caused by genetic variation is correlated with the treatment success in cancer and cardiovascular illness.

Direct applications that connect regulatory variation, miRNA-centered networks, and therapy response are starting to take shape. For drug repositioning, network analyses that integrate miRNA–mRNA regulatory architecture in cancer have identified repurposable compounds in lung cancer—illustrating a path for adding regQTL features next (Ye et al., 2023). On the immunotherapy side, germline polymorphisms in the PD-L1 3′-UTR (notably rs4143815) have been associated with outcomes under PD-1 blockade in NSCLC, supporting the idea that noncoding variants affecting post-transcriptional regulation can stratify checkpoint inhibitor benefit (Nomizo et al., 2017; Yoshida et al., 2021). For preclinical translation, patient-derived organoids already predict chemotherapy responses and clinical progression-free survival in colorectal cancer, providing a practical platform to functionally test genotype-informed (including regQTL-informed) treatment hypotheses (Ooft et al., 2019; Wang et al., 2023). Finally, advances in population-scale single-cell genetics show how regulatory effects can be mapped at cell-type resolution-an essential prerequisite for bringing regQTL signals (including miRNA-related ones) into mechanistically faithful, spatially and cellularly resolved models (van der Wijst et al., 2018; Yazar et al., 2022).

Together, these advances underscore the potential of regQTL-informed modeling to transform predictive oncology. By incorporating genetic, epigenetic, transcriptomic, and spatial data into deep learning frameworks, researchers are beginning to generate clinically relevant predictions that connect non-coding variation to therapeutic outcomes. As datasets expand and algorithms mature, regQTL-guided computational pharmacogenomics could become a cornerstone of precision cancer therapy.

### 5.5 miRNA-tailored immunotherapy and vaccine enhancement in cancer

miRNAs shape antitumor immunity at multiple levels tumor-intrinsic pathways, antigen presentation, and T-/NK-cell effector programs making them attractive levers to augment immunotherapy. Three complementary lines of evidence support this translational direction.

- miRNA modulation can potentiate immune-checkpoint blockade: In melanoma models, systemic delivery of miR-21-3p using gold nanoparticles enhanced anti-PD-1 efficacy by inducing tumor ferroptosis and increasing CD8^+^ T cell infiltration, providing a concrete demonstration that rational miRNA augmentation can sensitize tumors to checkpoint therapy (Guo et al., 2022).
- Noncoding germline variation at immune checkpoints associates with ICI outcomes: Polymorphisms in the PD-L1 (CD274) 3′UTR notably rs4143815 and related loci have been linked to response and survival in nivolumab-treated NSCLC, supporting the premise that inherited variants affecting post-transcriptional regulation can stratify checkpoint benefit (Nomizo et al., 2017) and have been further examined in larger retrospective series (Yoshida et al., 2021).
- Engineering dendritic-cell (DC) vaccines with miRNA control improves antitumor priming: In human monocyte-derived DCs, miR-155 enhances IL-12p70 secretion and augments NK/T cell activation by targeting SOCS1/KPC1/CD115, establishing a mechanistic handle for vaccine enhancement (Lu et al., 2011). Follow-up preclinical work using miR-155–overexpressing DC vaccines reported stronger antigen-specific T cell responses, suppressed tumor growth, and reduced metastasis in breast cancer models, underscoring feasibility of miRNA-programmed cellular vaccines (Hodge et al., 2020). Broader DC-vaccine engineering blueprints further outline how to combine miRNA rewiring with next-generation platforms to optimize antigen presentation and Th1 polarization (Perez and De Palma, 2019).

Together, these studies suggest two near-term strategies: (1) co-therapies that deliver miRNA mimics/antagomirs to reprogram tumor or myeloid compartments and thereby raise response rates to PD-1/PD-L1 or CTLA-4 inhibitors; and (2) cell-therapy upgrades, where *ex vivo* DCs (or other immune cells) are miRNA-engineered to optimize IL-12–driven Th1 polarization and durable cytotoxic immunity. In parallel, germline 3′UTR variants at immune regulators (e.g., PD-L1) merit prospective testing as predictive biomarkers for ICI benefit and toxicity. Moving from proof-of-concept to practice will require standardized delivery systems, on-target/off switch controls for safety, and multi-ethnic validation cohorts to ensure generalizability (Table 5).

**TABLE 5:** Validated regQTLs and their clinical/therapeutic relevance in cancer.

Gene/Variant (SNP–miRNA site)	Cancer Type(s)	Functional/Clinical effect	DOI
MDM4 rs4245739 (miR-191 site)	Breast, ESCC, ovarian	Creates novel miR-191 site; reduces MDM4 expression; associated with susceptibility and outcomes	Zhou et al. (2013)
KRAS rs61764370 (let-7 site)	Colorectal, NSCLC, HNSCC	Disrupts let-7 binding; linked to risk and therapy response (anti-EGFR)	Chen et al. (2008), Christensen et al. (2009)
SET8 (KMT5A) rs16917496 (miR-502 site)	NSCLC, ovarian	Alters miR-502 regulation; associated with overall survival	Xu et al. (2013)
LAMC1 rs3768617 (miR-548b-3p site)	Lung adenocarcinoma	T allele disrupts miRNA repression; increases LAMC1; promotes tumor growth; associated with poor prognosis	Yu et al. (2021)
PD-L1 3′UTR (rs4143815, rs2297136)	NSCLC, gastric	Variants alter miRNA binding (miR-570, miR-324); linked to PD-L1 expression, prognosis, and ICI response	(Lee et al., 2016; Yoshida et al., 2021)11/1/2025 3:52:00 p.m.
CD133/Prom1 (miR-135a/b site)	Lung cancer	Variant enhances repression; associated with favorable prognosis	Cheng et al. (2013)
ETS2 3′UTR SNP (miRNA site)	NSCLC	Affects miRNA regulation of ETS2; predictive for paclitaxel–cisplatin response	Hong et al. (2016)

## 6 Challenges, limitations, and future directions in miRNA-regQTL research

Research into regulatory quantitative trait loci (regQTLs) that influence miRNA–mRNA interactions has revealed new insights into cancer biology, yet several challenges limit their translational potential.

### 6.1 Incomplete annotation and limitations of classical sequence-based tools

The incomplete annotation of functional miRNA–target interactions is one of the major limitations. Several tools like TargetScan, miRanda, and RNAhybrid are heavily relying on canonical seed matching, however, they frequently miss non-canonical binding and generate false positive results. Recent research suggests that specificity of prediction models can be enhanced by incorporating structural accessibility. For instance, Kertesz et al. (2007) demonstrated that considering energy consumption of unpairing mRNA secondary structures could improve the miRNA target prediction accuracy (Kertesz et al., 2007). Similarly, recent study that assessed the experimental data (CLASH) has further stated the requirement of prediction frameworks that can integrated seed match and structural accessibility (Homberg et al., 2023).

### 6.2 Context dependency and cell-type specificity in regQTL effects

The context dependency and cell-type specificity also act as important barrier. Most of the studies on regQTL are rely on bulk tissue data and limit the cell-restricted regulatory events. Recent evidence showed that the effects of genetics on gene expression are mostly cell-type dependent, with some regulatory interactions detectable in specific immune or stromal compartments. For instance, recent study on cell-type interaction eQTLs (ieQTLS) in GTEx tissue have uncovers the hidden tissue specific regulatory variants that were missed in bulk profiling (Kim-Hellmuth et al., 2020). In addition, recent reviews of miRNA target prediction techniques also underline the importance of contextual elements such as expression levels of RNA-binding proteins and cell-type–specific miRNA expression patterns (Riolo et al., 2020). These studies further necessitate the use of scATAC-seq, spatial transcriptomics, and single-cell transcriptomics in future regQTL identification workflows.

### 6.3 Technical barriers and emerging solutions for single-cell regQTL analysis

Although single-cell technologies hold strong potential for dissecting regQTL activity at cellular resolution, current scRNA-seq platforms present significant limitations. Most droplet-based methods capture only short 3′transcript tags, providing insufficient coverage across the 3′UTR regions where the majority of miRNA-binding sites reside. This constraint severely limits the ability to identify allele-specific changes in miRNA–mRNA interactions. Moreover, high dropout rates, sparse transcript capture, and difficulties in quantifying low-abundance miRNAs add further uncertainty. These challenges are consistent with findings from cell-type–specific eQTL studies, where many regulatory associations remain undetected due to limited read depth and incomplete 3′UTR representation (van der Wijst et al., 2018).

Emerging technologies may help overcome these constraints. Full-length single-cell RNA-seq platforms such as SMART-seq3 significantly improve 3′UTR coverage and enable detection of isoform usage changes that influence miRNA-binding site accessibility (Hagemann-Jensen et al., 2020). Likewise, single-cell long-read sequencing using Oxford Nanopore or PacBio technologies allows reconstruction of complete transcript isoforms and identification of variant-bearing 3′UTRs at nucleotide resolution (Volden and Vollmers, 2022). Single-cell multi-omics approaches, including scATAC-seq and spatial transcriptomics, offer complementary layers to infer cell-type–specific regulatory mechanisms. Furthermore, perturbation-based single-cell assays such as Perturb-seq can test variant functionality by introducing allelic changes and tracking transcriptomic consequences across diverse cellular states (Replogle et al., 2020).

### 6.4 Population structure and ancestry bias

Population structure represents a major limitation in regQTL discovery, as most available datasets, including TCGA and GTEx are heavily enriched for individuals of European ancestry. This imbalance restricts the detection of ancestry-specific regQTLs, particularly for variants whose allele frequencies, linkage disequilibrium (LD) patterns, or transcript isoform usage differ substantially across populations. As a result, regulatory variants with functional importance in underrepresented groups may be missed entirely, while effect sizes derived from Eurocentric cohorts may not generalize to diverse clinical populations.

The implications of this bias are substantial. Genetic regulation of transcript abundance can vary markedly across ancestries, and studies such as Kim-Hellmuth et al. (2020) have demonstrated that eQTL effect sizes, tissue-sharing patterns, and interaction signals differ by population subgroup (Kim-Hellmuth et al., 2020). Given that miRNA expression profiles, RNA-binding protein abundance, and 3′UTR isoform distributions also show population-level variation, ancestry-specific differences may significantly alter miRNA–mRNA regulatory architecture. Consequently, failure to account for population structure may lead to false negatives, biased effect estimates, or even misclassification of clinically relevant regQTLs.

Addressing this limitation will require a concerted shift toward multi-ancestry regQTL discovery frameworks. Future studies should incorporate ancestry-informed genotype principal components, population-specific LD maps, and fine-mapping strategies calibrated across diverse groups. Integration of emerging multi-ethnic cohorts such as H3Africa (Choudhury et al., 2020), TOPMed (Taliun et al., 2021), gnomAD (Gudmundsson et al., 2022), and the All of Us Research Program (The All of Us Research Program Investigators, 2019) will be essential for expanding the catalog of ancestry-specific regQTLs and ensuring equitable clinical translation. Ultimately, improving ancestral representation is crucial not only for scientific accuracy but also for the development of regQTL-based biomarkers that are valid and effective across globally diverse patient populations.

### 6.5 Limited functional validation of regQTLs

A persistent challenge in miRNA-regQTL research is the limited availability of rigorous functional validation. Although biochemical assays such as CLIP-seq and CLASH have mapped thousands of putative miRNA–mRNA interactions, only a small fraction of predicted regQTLs have been experimentally tested. These assays confirm physical binding but do not determine whether a specific SNP alters the regulatory potency of miRNA-mediated repression. Recent work by Homberg et al. (2023) demonstrated that CLASH-derived interactions can improve target-prediction benchmarking; however, these data alone cannot establish allele-specific regulatory consequences in cancer (Homberg et al., 2023). To overcome this bottleneck, a structured and multi-layered validation framework is required.

#### 6.5.1 Reporter-based validation of allele-specific effects

Luciferase and GFP/RFP reporter assays enable controlled testing of SNP-modified miRNA-binding sites by quantifying changes in repression efficiency between reference and alternate alleles. These assays remain the gold standard for validating direct miRNA–mRNA regulatory effects (Gao et al., 2015).

#### 6.5.2 Genome editing to test variants in endogenous contexts

CRISPR/Cas9 editing, including base editing and prime editing allows precise introduction of regQTL alleles into native genomic loci, enabling evaluation of allele-specific regulatory outcomes within physiologically relevant chromatin and transcriptional environments (Dangelmaier et al., 2022).

#### 6.5.3 High-throughput perturbational screening

Massively parallel reporter assays (MPRAs) provide a scalable platform for testing thousands of miRNA-binding variants simultaneously, generating large-scale quantitative maps of allele-dependent regulatory effects (Vainberg Slutskin et al., 2018; Dejnirattisai et al., 2021).

#### 6.5.4 Single-cell and multi-omics functional pipelines

Emerging platforms such as Perturb-seq and CRISPR-scRNA-seq enable evaluation of allelic perturbations at single-cell resolution, revealing cell-type–specific regulatory outcomes that are often missed in bulk assays. Integration with scATAC-seq, spatial transcriptomics, and miRNA-seq further permits characterization of how regQTLs reshape regulatory networks within heterogeneous tumor microenvironments (Adamson et al., 2016; Dixit et al., 2016; Wu et al., 2017; Replogle et al., 2020; Prajapat et al., 2025).

#### 6.5.5 *In vitro* and *in vivo* modeling of regQTL effects

Organoid systems, xenograft models, and patient-derived cell lines provide intermediate-to-high biological fidelity platforms for validating functional variants under physiologically relevant conditions. These systems are essential for linking regQTL activity to phenotypes such as proliferation, metastasis, immune evasion, and drug response (Hidalgo et al., 2014; Clevers, 2016).

Together, these complementary approaches underscore the need for a systematic and scalable validation pipeline. Without rigorous allele-specific confirmation, most computationally identified regQTLs remain putative and cannot be confidently incorporated into mechanistic models or translated into clinical biomarker frameworks. Closing this validation gap is therefore one of the most critical future directions for the field.

### 6.6 Future directions and translational outlook

Another limitation associated with regQTLs research is their clinical translational ability. Even though hundreds of studies have examined diagnostic and predictive potential of miRNA, many findings remain difficult to interpret due to germline mutations that modify miRNA–mRNA interactions. Furthermore, consistent framework for integration of regQTLs into regulatory-grade biomarker panels is still lacking that further restrict clinical applicability. Thus, therapeutic value regQTLs is still understudied in comparison to other well-known indicators such gene expression patterns and somatic mutations.

Moving forward, the field required a standardized regQTL identification pipeline that incorporate sequence, structure, RNA-binding protein interactions, and biochemical binding data (e.g., AGO-CLIP, CLASH). Expansion of functional assays including CRISPR-based allele editing, luciferase reporter systems, and high-throughput MPRAs, will be essential to generate the evidence base needed for clinical deployment. Likewise, as multi-omic profiling becomes routine in translational oncology that integrate regQTLs into composite biomarker frameworks will be critical for capturing their contribution to tumor behavior, treatment response, and inter-individual variability.

Future research should also prioritize population-specific regQTL mapping, single-cell resolution analyses, and integration with proteomic and metabolomic data to better characterize the dynamic behavior of miRNA regulatory networks under therapeutic pressure and evolving tumor microenvironments. Importantly, incorporating regQTL information into clinical trial design—including trials for miRNA-based immunotherapies, antisense oligonucleotide therapeutics, and mRNA vaccines—may substantially improve patient stratification and treatment personalization. By systematically validating functional variants and linking them to drug response mechanisms, regQTL research has the potential to accelerate the development of next-generation precision oncology interventions.

Finally, meaningful clinical translation will depend on ensuring ancestry-inclusive discovery. Programs such as H3Africa and All of Us provide an important foundation for identifying population-specific regQTLs and preventing Eurocentric bias in biomarker development. Establishing regulatory guidance and data standards for evaluating regQTL-based diagnostics will be paramount to enabling their eventual adoption in precision oncology.

## 7 Conclusion

The expanding field of regulatory QTLs (regQTLs), with a specific focus on microRNA (miRNA)–mediated regulation, is reshaping our understanding of post-transcriptional gene regulation in cancer. Unlike traditional eQTLs, which assess steady-state mRNA abundance, regQTLs capture a dynamic layer of regulation by identifying germline variants that alter miRNA–mRNA interaction strength. These variants can disrupt or enhance miRNA-mediated repression, influencing oncogenic signaling, tumor progression, immune evasion, and therapeutic resistance. The discovery of candidate regQTLs has increased substantially with high-throughput sequencing, single-cell technologies, and large-scale biobanks, enabling the detection of variants with measurable regulatory effects across diverse cancer types.

Throughout this review, we summarized several computational strategies used to prioritize functional variants. These include transcriptome-wide association studies (TWAS), allele-specific miRNA binding prediction tools, and miR-eQTL analyses, which function as complementary upstream approaches for identifying candidate miRNAs and regulatory variants that may participate in downstream regQTL interactions. When combined with direct regQTL modeling and supported by experimental assays such as luciferase reporters, CRISPR-based editing, and AGO-CLIP, these methods form a robust framework for identifying causal SNPs that modify miRNA-mediated gene regulation. Importantly, several regQTLs have been linked to clinically meaningful outcomes, including drug response, patient survival, and immune phenotypes, underscoring their translational relevance.

Despite these advances, key challenges remain. The context-dependence of miRNA–mRNA interactions complicates interpretation, and the limited number of experimentally validated variants restricts confidence in many predicted associations. Current datasets remain enriched for individuals of European ancestry, limiting global applicability. Moreover, miRNA regulatory networks are dynamic and responsive to microenvironmental and therapeutic cues, which poses challenges for static modeling approaches. Moving forward, integration of regQTLs with multi-omic datasets—including epigenomic, proteomic, and metabolomic profiles—will be essential for capturing the full scope of their mechanistic impact. Functional high-throughput assays will remain critical for validating candidate variants, and greater ancestral diversity will strengthen the generalizability and equity of regQTL-based discoveries. Incorporating regulatory variants into clinical trial design may further bridge the gap between genomic discovery and precision oncology.

In conclusion, regQTLs that modulate miRNA function and targeting represent an important interface between germline genetics and tumor biology. Continued methodological, experimental, and clinical advances will enable their systematic integration into biomarker development, prognostic modeling, and therapeutic decision-making, positioning regQTLs as a meaningful and underutilized component of precision oncology.
